# Long non-coding RNA Loc490 inhibits gastric cancer cell proliferation and metastasis by upregulating RNA-binding protein Quaking

**DOI:** 10.18632/aging.103876

**Published:** 2020-09-15

**Authors:** Zhengxi He, Zhaojun Duan, Ling Chen, Bin Li, Yanhong Zhou

**Affiliations:** 1 Department of Oncology, Xiangya Hospital, Central South University, Changsha 410008, People’s Republic of China; 2 Basic School of Medicine, Cancer Research Institute, Central South University, Changsha 410008, Hunan, People’s Republic of China; 3 Hunan Cancer Hospital, The Affiliated Tumor Hospital of Xiangya Medical College, Central South University, Changsha 410008, People’s Republic of China; 4 Key Laboratory of Carcinogenesis of the Ministry of Health and Key Laboratory of Carcinogenesis and Cancer Invasion of the Ministry of Education, Cancer Research Institute, Central South University, Changsha 410008, People’s Republic of China; 5 Medical Research Center, Key Laboratory of Cancer Proteomics of the Chinese Ministry of Health, Xiangya Hospital, Central South University, Changsha 410008, People’s Republic of China; 6 Department of Gastrointestinal Surgery, Xiangya Hospital, Central South University, Changsha 410008, People’s Republic of China; 7 National Clinical Research Center for Geriatric Disorders, Xiangya Hospital, Central South University, Changsha 410008, People’s Republic of China

**Keywords:** gastric cancer, long non-coding RNA Loc490, RNA-binding protein QKI, epithelial-mesenchymal transition, lymph node metastasis

## Abstract

Gastric cancer (GC) is one of the most common malignant tumor types worldwide. Long non-coding RNAs (lncRNAs) have important epigenetic effects, including altering the proliferation and metastasis of malignant tumors. We used gene chip technology to search for lncRNAs that were differentially expressed in GC and metastatic lymph node tissues compared with adjacent normal tissues. The lncRNA Loc490 and the RNA-binding protein Quaking (*QKI*) were downregulated in GC tissues and lymph node metastases compared with normal tissues, and the levels of these two genes correlated positively with one another. Loc490 expression correlated negatively with lymph node metastasis and vein/nerve invasion, while it correlated positively with overall and disease-free survival. *In vitro*, Loc490 post-translationally enhanced the expression of QKI and suppressed the expression of epithelial-mesenchymal transition-related molecules. Overexpression of Loc490 inhibited GC cell proliferation, invasion and metastasis and exerted strong antitumor effects *in vivo*, while silencing of *QKI* antagonized these effects. A potential binding site between Loc490 and QKI was detected through bioinformatics analysis and confirmed through RNA immunoprecipitation and mutant analyses. Our results suggest that lncRNA Loc490 inhibits GC cell proliferation and metastasis by upregulating RNA-binding protein QKI.

## INTRODUCTION

Gastric cancer (GC), known for its lymph node metastasis and high morbidity and mortality, is one of the most common malignant tumors in Southeast Asia, Central/Eastern Europe and Africa. Although the incidence and mortality of GC have declined gradually over the years, GC is still the second leading cause of cancer-related death worldwide due to the lack of early-stage diagnostic methods and effective treatments. In 2015, there were 679,100 new GC cases and 498,000 GC-related deaths in China, making GC second only to lung cancer [1].

The size, infiltration depth, lymph node metastasis and distant metastasis rates of GC are important prognostic indicators. Various mechanisms may contribute to the malignant proliferation and metastasis of GC cells, including the epithelial-mesenchymal transition (EMT). During the EMT, epithelial cells acquire mesenchymal characteristics such as extracellular matrix degradation, increased activity and anti-apoptotic properties, thereby becoming more malignant [2–4]. Thus, elucidating the regulatory mechanisms of the EMT is vital for preventing and treating malignant tumors.

Long non-coding RNAs (lncRNAs) are non-coding RNAs with > 200 base pairs, which were once thought to be transcriptional noise, but are now known to be key regulators of epigenetics, the cell cycle and differentiation [5]. Abnormal expression of various lncRNAs contributes to the development of malignant tumors [6, 7], including GC. For instance, lncRNA HOTAIR promotes GC cell invasion, migration and chemotherapy resistance by activating the phosphoinositide 3-kinase/AKT/multidrug resistance-associate protein 1 and Wnt/β-catenin pathways and upregulating Human epidermal growth factor receptor 2 [8–10]. LncRNA H19, the earliest discovered imprinted gene, is upregulated in GC tissues and enhances the malignant proliferation of GC via a novel pathway H19/miR-675/RUNX1 but also directly up-regulate the expression of ISM1 and indirectly inhibit the expression of CALN1 through miR-675, which can potentially be contributes to the malignant biological behaviour of GC cells [11–13]. LncRNA metastasis-associated lung adenocarcinoma transcript 1 enhances EMT-induced GC cell invasion and migration [14]. LncRNA maternally expressed gene 3 is downregulated in GC tissues and can suppress GC proliferation and metastasis by upregulating P53 and downregulating the transcription factor E2F3 [15–16].

The above studies have indicated that lncRNAs are involved in the progression of GC; however, the functions of the majority of lncRNAs have not been fully elucidated. In the present study, we identified an additional lncRNA that was differentially expressed in GC tissues and explored its mechanism of action.

## RESULTS

### Expression of Loc490 and *QKI* in GC-associated tissues

**Figure 1:**
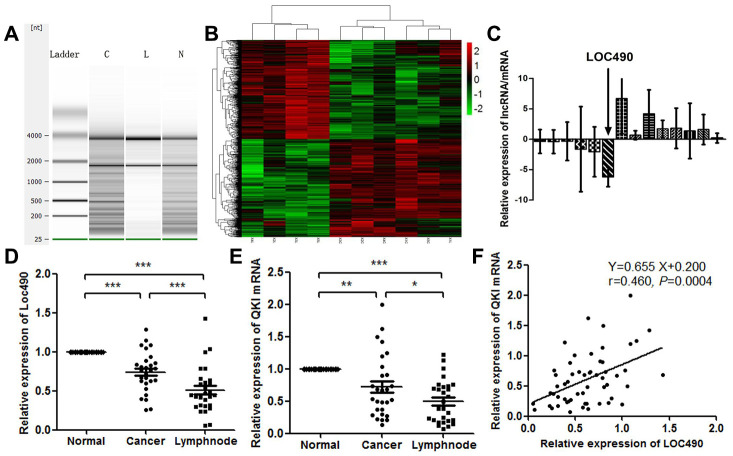
**Low expression of Loc490 in GC and lymph node metastasis tissue.** (**A**) RNA quality testing (C: GC tissue; L: lymph node metastasis tissue; N: normal tissue adjacent to GC); (**B**) Screening for differentially expressed lncRNAs using Agilent Human LncRNA chip technology in normal tissues adjacent to GC, primary GC tissues and lymph node metastasis tissues; (**C**) Loc490 expression was significantly lower in lymph node metastases than in normal tissues adjacent to GC and in primary GC tissue (the other selected genes are shown in the article); (**D**) qRT-PCR results demonstrating Loc490 expression in 28 normal tissues adjacent to GC, primary GC tissues and lymph node metastasis tissues; (**E**) Expression of *QKI* mRNA in 28 normal tissues adjacent to GC, primary GC tissues and lymph node metastases; (**F**) Correlation analysis demonstrating that Loc490 expression was significantly positively correlated with *QKI* mRNA expression. Data represent the mean and SD from three experiments. **p < 0.01, ***p < 0.001.

We used an Agilent Human LncRNA gene chip to compare the lncRNA and mRNA levels in GC and metastatic lymph node tissues with those in normal tissues adjacent to the GC. In total, 2669 lncRNAs were upregulated and 3506 lncRNAs were downregulated (fold change > 2.0) in GC tissues and lymph node metastasis tissues compared with adjacent normal tissues. To determine the correlation of aberrantly expressed lncRNAs with the metastatic phenotype in GC, we compared the expression profiles of GC samples, adjacent normal tissues and lymph node metastases after ensuring that the RNA was not degraded (Figure 1A and 1B). Upon repeating the analysis and narrowing the results by gene expression consistency, we found that 1707 lncRNAs were differentially expressed in these samples, and there were more upregulated lncRNAs (1025) than downregulated lncRNAs (682) in the cancerous tissues. The consistently abnormally expressed lncRNAs correlated with lymph node metastasis, distant metastasis and advanced tumor-node-metastasis (TNM) stages.

To validate the microarray data, we used a Pearson algorithm to calculate the correlation between mRNA and lncRNA levels. Based on their fold-changes, the nine the most differentially expressed lncRNAs (upregulated lncRNAs: CD4, PRPF40B, SRGN, FGD2, C17orf46 and ZBTB; downregulated lncRNAs: LOC101927490, LOC101929897 and C8orf49) were selected from the aberrantly expressed lncRNAs. We also selected the five most differentially expressed mRNAs (upregulated mRNAs: *TGFβ-1* and *FXYD5*; downregulated mRNAs: *MUC5AC*, *MUC6* and *TMEM97*). Then, we validated the differential expression of the selected lncRNAs in 83 clinical tissue samples using quantitative real-time PCR (qRT-PCR) (Figure 1C). Despite great variability due to the small sample size, the fold-changes in all the lncRNAs in the 83 clinical samples were similar to those measured on the microarray. LOC101927490 (Loc490) was stably downregulated in GC tissues and metastatic lymph nodes compared with adjacent normal tissues. The average levels of all the selected lncRNAs differed between tissues with and without lymph node metastases (p < 0.01 or p < 0.05 for specific lncRNAs). These results revealed the consistency between the qRT-PCR data and the microarray data.

Next, we performed a bioinformatics analysis to identify Loc490-binding proteins. The RNA-binding protein Quaking (QKI) was retrieved. QRT-PCR analysis of the collected clinical samples revealed that the expression pattern of *QKI* was consistent with that of Loc490 (Figure 1D and 1E). This finding was confirmed in a correlation analysis (Figure 1F).

### Correlation between Loc490 expression and patients’ clinical pathological data and prognoses

Next, we examined the correlation between lncRNA Loc490 expression and patients’ clinical pathological features. As shown in Table 1, there were no differences in age, gender, histologic grade or carcinoembryonic antigen levels according to Loc490 expression. However, low expression of Loc490 was associated with a pathological metastasis status and distal lymph node metastasis (p < 0.05) and correlated with TNM staging (although the p value was not statistically significant), suggesting that Loc490 expression probably correlated negatively with metastasis status and positively with overall survival.

**Table 1:** The expression of Loc490 and clinical parameters of patients with GC (n=83).

**Characteristics**	**Loc490 Expression Levels**		**P value**
	**Low (n,%)**	**High (n,%)**	
*Gender*			
Male	37 (44.6%)	23 (27.7%)	0.57
Female	14 (16.9%)	9 (10.8%)	
*Age (years)*	66.9 ± 11.3	64.5 ± 11.4	0.463
*Histology*			
Well, moderate	11 (13.2%)	13 (15.7%)	0.083
Poor, signet	40 (48.2%)	19 (22.9%)	
*Depth*			
m, sm, mp	10 (12%)	9 (10.8%)	0.426
ss, se, si	41 (49.4%)	23 (27.7%)	
*Lymph node invasion*			
Yes	36 (43.4%)	18 (21.7%)	0.238
No	15 (18.1%)	14 (16.9%)	
*Venous invasion*			
Yes	39 (47%)	17 (20.5%)	0.033*
No	12 (14.5%)	15 (18.1%)	
*Nervous invasion*			
Yes	35 (42.2%)	13 (15.7%)	0.022*
No	16 (19.3%)	19 (22.9%)	
*Lymph node metastasis*			
Yes	24 (28.9%)	7 (8.4%)	0.035*
No	27 (32.5%)	25 (30.1%)	
*TNM stage*			
I–II	12 (14.5%)	14 (16.9%)	0.088
III–IV	39 (47%)	18 (21.7%)	
*CEA (μg/ml)*			
≤2.5	18 (21.7%)	15 (18.1%)	0.359
>2.5	33 (39.8%)	17 (20.5%)	

**Figure 2:**
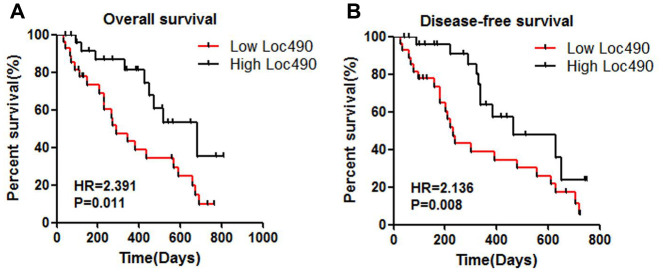
**Low expression of Loc490 indicates poor prognosis of GC.** (**A**) Overall survival; (**B**) Disease-free survival.

Univariate and multivariate analyses were performed to determine which clinical variables were associated with the survival of GC patients. Although Loc490 expression was significantly associated with survival in the univariate analysis, it did not independently predict overall survival in the multivariate analysis. On the other hand, distal metastasis coupled with the TNM stage was strongly associated with overall survival in the multivariate Cox proportional hazards model. A Kaplan-Meier survival analysis and log-rank test were also used to evaluate the effects of Loc490 expression and clinical pathological characteristics on overall survival. The results indicated that patients with low Loc490 levels had significantly worse overall survival and disease-free survival than patients with high Loc490 levels (Figure 2).

### Loc490 expression in GC cell lines and effects of Loc490 overexpression/knockdown

**Figure 3:**
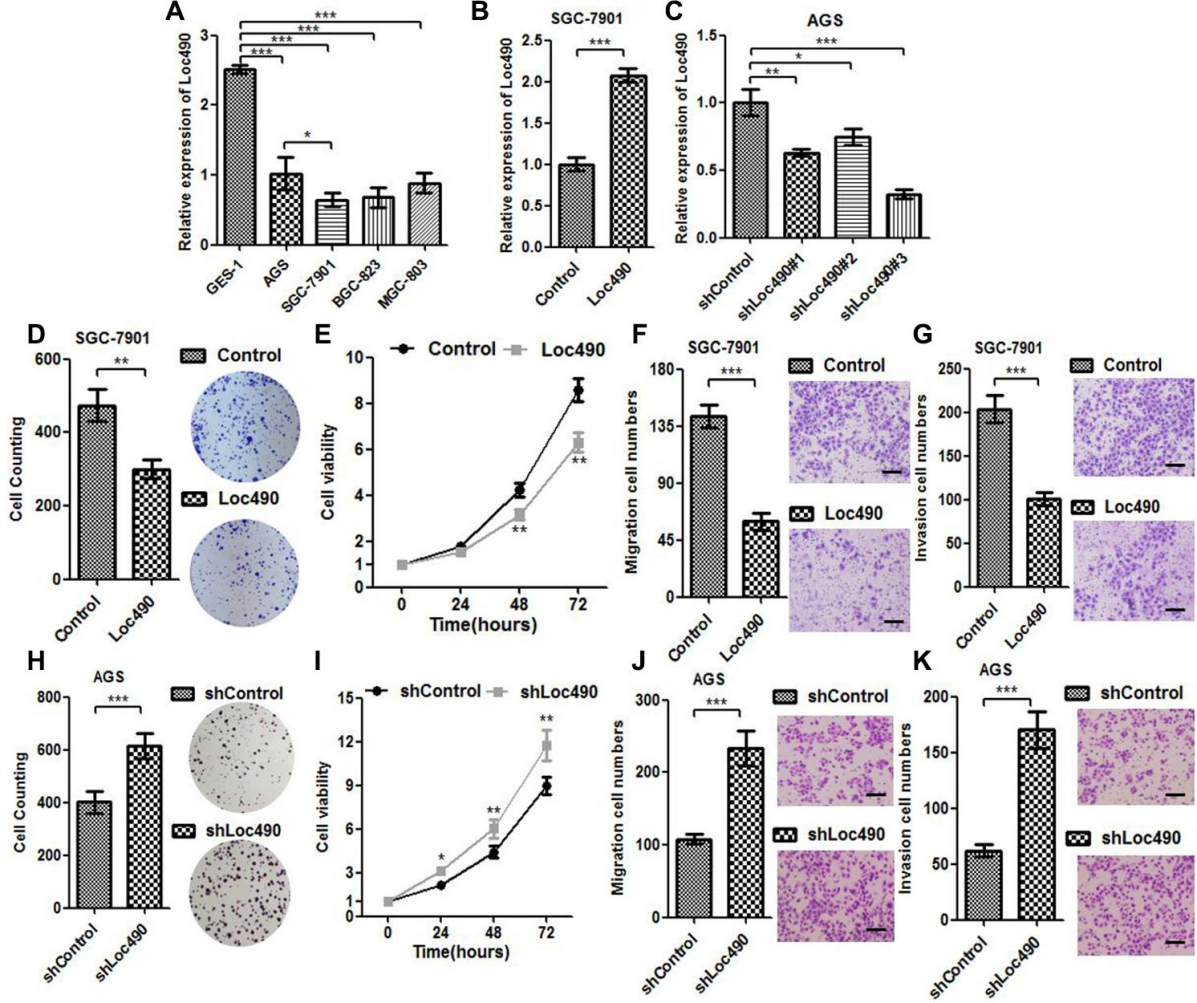
**Low expression of Loc490 confers GC cell proliferative advantage.** (**A**). Expression of Loc490 in a normal gastric epithelial cell line and GC cell lines; (**B**, **C**) Overexpression/knockdown of Loc490 in GC cell lines; (**D**, **H**) Clone formation results in Loc490-overexpressing/knockdown cell lines; (**E**, **I**) MTS assay results; (**F**, **J**) Invasion assay results and quantification; (**G**, **K**) Migration assay results and quantification. Scale bars: 100 μm. Data represent the mean and SD from three experiments. *p < 0.05, **p < 0.01, ***p < 0.001.

We next used qRT-PCR to assess Loc490 expression in different GC cell lines (AGS, SGC-7901, BGC-823 and MGC-803) and normal gastric epithelial cells (GES-1). Loc490 levels were lower in the GC cell lines than in GES-1 cells (Figure 3A). Among the GC cell lines, SGC-7901 cells exhibited the lowest Loc490 levels, while AGS cells exhibited the highest levels. Thus, to determine the significance of Loc490 downregulation in GC tissues, we constructed Loc490 overexpression and interference vector plasmids and transfected them into SGC-7901 and AGS cells, respectively. QRT-PCR analysis confirmed the overexpression and knockdown of Loc490 in the transfected GC cell lines (Figure 3B and 3C).

Next, we performed MTS (3-(4,5-dimethylthiazol-2-yl)-5-(3-carboxymethoxyphenyl)-2-(4-sulfophenyl)-2H-tetrazolium), Transwell and clone formation assays to investigate the effects of overexpressing or silencing Loc490 on the invasion and metastasis of GC cell lines, using an empty plasmid cell line as the control group (Figure 3D–3H). The number of migrating and invading GC cells was significantly lower in the Loc490 overexpression group than in the control group, while it was significantly greater in the Loc490 knockdown group than in the control group. Thus, the expression of lncRNA Loc490 correlated inversely with the invasion and metastasis abilities of GC cells, consistent with the results of the previous experiments.

### Effects of Loc490 on QKI, N-cadherin, E-cadherin, MMP-9 and Vimentin expression

**Figure 4:**
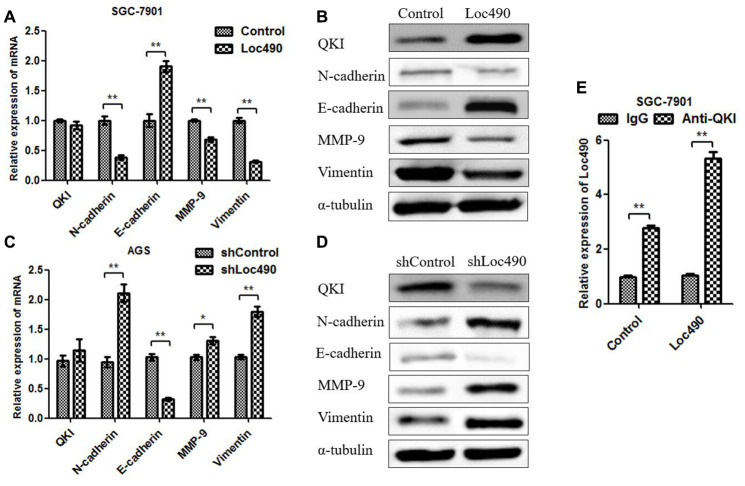
**The expression of Loc490 changes the expression of EMT-related proteins.** (**A**, **C**) Changes in *QKI*, *N-cadherin*, *E-cadherin*, *MMP-9* and *Vimentin* mRNA levels in Loc490-overexpressing cell lines; (**B**) Changes in QKI, N-cadherin, E-cadherin, MMP-9 and Vimentin protein levels in Loc490-overexpressing cell lines; (**D**) Changes in QKI, N-cadherin, E-cadherin, MMP-9 and Vimentin protein levels in Loc490-knockdown cell lines; (**E**) RNA immunoprecipitation assay to detect Loc490 expression. Data represent the mean and SD from three experiments. *p < 0.05, **p < 0.01.

*QKI* mRNA levels were found to correlate with Loc490 levels in GC tissues. To further investigate the mechanism whereby Loc490 overexpression inhibited GC invasion and metastasis, we used qRT-PCR and Western blotting to detect QKI and invasion/metastasis-associated proteins in Loc490-overexpressing and - knockdown cell lines (Figure 4A–4D). N-cadherin, matrix metalloproteinase 9 (MMP-9) and Vimentin were downregulated in Loc490-oxerexpressing SGC-7901 cells, but were upregulated in Loc490-knockdown AGS cells. Interestingly, QKI and E-cadherin were upregulated in Loc490-overexpressing SGC-7901 cells, but were downregulated in Loc490-knockdown AGS cells. The mRNA and protein levels of N-cadherin and E-cadherin were consistent with one another in both cell types. These results suggested that the downregulation of Loc490 promoted the invasion and migration of GC cell lines by inducing invasion/metastasis-related proteins such as MMP-9 and Vimentin, while the overexpression of Loc490 inhibited tumor invasion by inducing QKI and E-cadherin.

### Binding of Loc490 to QKI suppresses GC invasion and metastasis *in vitro* and *in vivo*

**Figure 5:**
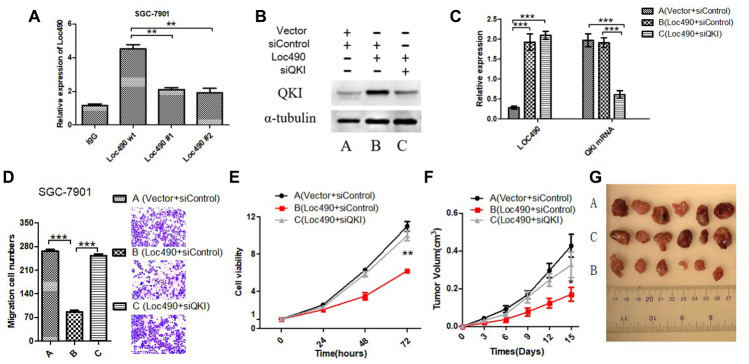
**Inhibit QKI expression by interfering Loc490 expression in GC cells and affect the biological behavior.** (**A**) Loc490 mutations at two binding sites; (**B**, **C**) Expression of Loc490 and QKI; (**D**, **E**). Invasion and proliferation assay results in SGC-7901 cells; (**F**) Tumor volume in each group of nude mice; (**G**) Representative tumors from each group are shown after three weeks (time calculated one week after the subcutaneous inoculation of the nude mice). (A: Vector + siControl, B: Loc490 + siControl, C: Loc490 + siQKI). Scale bars: 100 μm. Data represent the mean and SD from three experiments. **p < 0.01, ***p < 0.001.

To explore whether RNA-binding protein QKI could bind directly to Loc490, we performed a bioinformatics analysis, which revealed two potential binding sites on Loc490: nucleotides 760-778 UCCUAACCUG and 860-869 UAAUAACACA. Then, we mutated these two binding sites on Loc490 and used RNA-binding protein immunoprecipitation antibodies to detect the binding between the lncRNA and the protein. The binding of Loc490 to QKI decreased after the two binding sites on Loc490 were mutated, suggesting that QKI binds directly to these two sites (Figure 5A). Thus, Loc490 likely suppressed GC cell invasion and migration by binding to QKI, rather than performing various functions in parallel (Supplementary Figure 1).

To confirm that the effects of Loc490 in GC cells depended on the induction of QKI, we divided SGC-7901 cells into three groups for small interfering RNA (siRNA) experiments: Vector + control siRNA (siControl), Loc490 + siControl, and Loc490 + *QKI* siRNA (siQKI*)*. QRT-PCR and Western blotting demonstrated that the siRNA effectively inhibited *QKI* expression (Figure 5B and 5C). Then, the invasion and metastasis abilities of the transfected cells were detected with MTS and Transwell assays (Figure 5D and 5E). In addition, nude mice were subcutaneously inoculated with the transfected SGC-7901 cells, and their tumor volumes were measured (Figure 5F and 5G). The viability and invasiveness of the cells and the tumor volumes of the mice were significantly lower in the Loc490 + siControl group than in the Vector + siControl group and the Loc490 + siQKI group. These results indicated that the invasion and metastasis abilities of GC cells decreased when Loc490 was overexpressed, consistent with our previous experimental results (Figure 3). In summary, we confirmed that the direct binding of lncRNA Loc490 to RNA-binding protein QKI inhibited the proliferation and metastasis of GC *in vitro* and *in vivo*.

## DISCUSSION

QKI, which belongs to the Signal Transduction and Activation of RNA family of KH domain-containing RNA-binding proteins, participates in pre-mRNA splicing, microRNA regulation and circular RNA formation [17]. Many studies have revealed that QKI is a crucial tumor suppressor that prevents the initiation and progression of various malignancies. For instance, lower QKI expression in lung cancer was associated with a poorer prognosis, while QKI overexpression inhibited the malignant proliferation of lung cancer cells *in vivo* and *in vitro* by preventing Notch signaling pathway activation [18]. QKI expression was reduced in prostate cancer tissue and was associated with cell differentiation, the TNM stage and the overall survival rate. Accordingly, QKI overexpression inhibited prostate cancer cell proliferation and tumorigenesis *in vitro* and *in vivo* [19]. QKI was also downregulated in colorectal cancer tissues and cell lines, and was reported to inhibit cell cycle progression and invasion in colorectal cancer by negatively regulating microRNA-155 [20]. Low QKI expression was detected in oral cancer tissues and oral cancer stem cells, but cell and mouse experiments indicated that QKI could inhibit cancer stem cell sphere formation by binding to and inhibiting the 3' untranslated region of *SOX2* [21]. Lipopolysaccharide reduced QKI expression in a microRNA-155-dependent manner during leukemogenesis, whereas QKI overexpression impaired the lipopolysaccharide-induced phosphorylation of c-Jun N-terminal kinase and p38 mitogen-activated protein kinase [22]. The downregulation of QKI due to promoter hypermethylation was detected in GC tissues and correlated with a poorer differentiation status, greater invasion depth, gastric lymph node metastasis, distant metastasis, advanced TNM stage and poorer patient survival. However, QKI overexpression reduced the proliferation of GC cell lines *in vitro* [23].

Considering the extensive biological functions of lncRNAs and their importance in malignant tumors [24–26], we used a high-throughput sequencing technique to identify lncRNAs associated with GC metastasis. LncRNA Loc490 downregulation was found to be associated with GC metastasis, and a bioinformatics website (http://rbpdb.ccbr.utoronto.ca/) detected two potential binding sites (UCCUAACCUG and UAAUAACACA) between Loc490 and the anti-oncogene QKI. Furthermore, we assessed the expression of Loc490 and *QKI* and their relationship in clinical tissues. Both Loc490 and *QKI* were expressed at significantly lower levels in GC tissues and lymph node metastasis tissues than in adjacent normal tissues. These results suggested that the downregulation of Loc490 and *QKI* promotes the carcinogenesis and metastasis of GC. The positive correlation between Loc490 and *QKI* levels in tumor tissues further suggested that there could be a relationship between Loc490 and QKI.

We then analyzed the clinical significance of Loc490 in GC, and found that low Loc490 expression was associated with vein/nerve invasion, lymph node metastasis and a poor prognosis in GC patients. To clarify the effects of Loc490 on the malignant behavior of GC cells, we measured Loc490 expression in various GC cell lines. We found that Loc490 expression was lower in GC cell lines than in normal gastric epithelial cells. Since Loc490 expression was the lowest in SGC-7901 cells and highest in AGS cells, we used these cell lines to construct the Loc490 overexpression and interference cell models, respectively. The proliferation and metastasis abilities of SGC-7901 cells were significantly reduced after Loc490 overexpression, while the proliferation and metastasis abilities of AGS cells were significantly elevated after Loc490 silencing. Thus, reducing the expression of Loc490 promoted the proliferation and metastasis of GC cells *in vitro*.

To determine the impact of Loc490 on QKI and its downstream target genes, we detected the mRNA and protein levels of QKI and EMT-associated molecules in Loc490-overexpressing and -knockdown cells. N-cadherin, E-cadherin, MMP-9 and Vimentin are generally considered to be EMT-related proteins, and their expression is known to be associated with QKI expression [27–29]. Our results demonstrated that Loc490 significantly enhanced QKI protein expression and inhibited EMT-related protein expression. However, Loc490 did not significantly modify *QKI* mRNA levels, suggesting that Loc490 may alter the stability of QKI post-translationally, thus inducing QKI protein accumulation and downstream target gene expression. In addition, an RNA immunoprecipitation assay confirmed that Loc490 could bind to QKI protein. To better understand the upstream/downstream relationship between Loc490 and QKI, we used siRNA to reduce *QKI* expression in Loc490-overexpressing cells. We found that siQKI significantly antagonized the antitumor effects of Loc490 overexpression both *in vitro* and *in vivo*. Thus, Loc490 inhibits GC progression through its downstream effects on QKI. We also assessed potential binding sites between Loc490 and QKI, and found that mutating nucleotides 760-778 and 860-869 on Loc490 significantly reduced its ability to bind to QKI.

In conclusion, we performed transcriptome microarray analyses to detect differentially expressed lncRNAs in GC samples, normal stomach tissues and lymph node metastases. We found that the downregulation of lncRNA LOC101927490 was associated with increased TNM stages, enhanced lymph node metastasis and reduced overall survival in GC patients. Our *in vitro* experiments suggested that Loc490 post-translationally modifies QKI. In future studies, we will use post-translational modification proteomics to explore potential post-translational modification sites on QKI and to clarify the effects of Loc490 expression on them. Moreover, our *in vivo* studies indicated that Loc490 inhibited the subcutaneous tumorigenesis of GC cells in nude mice in a QKI-dependent manner (Figure 5F). Further studies are needed to explore this mechanism *in vivo* and to confirm the suitability of Loc490 as a new target for the clinical diagnosis and treatment of GC. We plan to investigate the effects of small molecules such as small-activating RNAs [30–31] on GC cells, to determine whether inducing Loc490 could antagonize the malignant behavior of these cells and delay or even prevent the clinical progression of GC.

## MATERIALS AND METHODS

### Cell culture, antibodies, plasmids and transfection

The human embryonic kidney cell line 293T, the normal gastric epithelial cell line GES-1 and the GC cell lines AGS, SGC-7901, BGC-823 and MGC-803 were obtained from the Cancer Research Institute of Central South University. Dulbecco’s modified Eagle’s medium/F12 (Hyclone) was used to culture the AGS cells. RPMI-1640 medium (Gibco) was used to culture the GES-1, SGC-7901, BGC-823 and MGC-803 cells. All the media were supplemented with 10% fetal bovine serum (FBS). The cell lines were negative for mycoplasma contamination. All cell lines were passaged less than 10 times after their initial revival from frozen stocks, and were authenticated via short tandem repeat profiling prior to use.

The primary antibodies against E-cadherin (BS90443) and N-cadherin (BS72312), along with the goat anti-rabbit IgG (H+L) horseradish peroxidase antibody (BS10003), were purchased from Bioworld. Antibodies against Vimentin (EPR3776), MMP-9 (EP1255Y) and QKI (EPR7306) were obtained from Abcam. Antibodies against α-Tubulin (T9026) were purchased from Sigma.

The lentiviral overexpression vector containing full-length Loc490 cDNA in a pLVX-EF1a-IRES-Puro plasmid (632186) was purchased from Clontech, along with the control vectors. The lentiviral short-hairpin RNA clones targeting Loc490 (STK300-10) and the non-targeting control vector (LP105-100) were purchased from GeneCopoeia. The Loc490 mutant #1 and #2 vectors were designed by and purchased from OE Biotech. The plasmid vectors were verified through sequencing.

We generated stable overexpression and knockdown cell lines by using Lipofectamine 2000 (Thermo Scientific, 11668027) to transfect 293T cells with pCMV-VSV-G (#8454), pMDLg-Prre (#12251) and pRSV-Rev (#12253) (Addgene) vectors containing the full-length Loc490, short-hairpin RNA targeting Loc490 and the corresponding controls, respectively. Viral supernatant fractions were collected 48 hours after transfection, and were filtered through a 0.45-μm filter (Millipore) before being used to infect GC cell lines three times over 24 hours. After three days, the cells were incubated in medium containing 1 μg/mL puromycin (Sigma) for one week.

### Study cohort and tissue samples

Between January 2014 and December 2015 at Xiangya Hospital, Central South University, 83 patients diagnosed with GC were randomly selected as cohort participants at random intervals. Among patients diagnosed with primary GC, those who received neoadjuvant chemotherapy treatment prior to surgery were excluded from the study. Every three months, each patient’s information was updated through a follow-up scheme or telephone visit. In total, 28 GC tissues and 28 corresponding adjacent normal tissues that were ≥ 5 cm from the cancer tissues were resected, placed in 1.5-mL Eppendorf tubes and stored at -80°C. The ethics committee of Xiangya Hospital, Central South University approved this study.

### Agilent Human LncRNA chip

An Agilent Human LncRNA gene chip (4*180K, Design ID: 076500) was used to detect the lncRNA levels in 15 clinical samples (5 normal tissues adjacent to GC, 5 GC tissues and 5 metastatic lymph node tissues). Total RNA was quantified on a NanoDrop ND-2000 (Thermo Scientific), and the RNA integrity was detected on an Agilent Bioanalyzer 2100 (Agilent Technologies). The marking of samples, hybridization of chips and elution were performed in accordance with standard procedures. Feature Extraction (version 10.7.1.1, Agilent Technologies) and Genespring (version 13.1, Agilent Technologies) were used to process the data and screen for differentially expressed genes (fold change > 2.0 and p value < 0.05). Gene Ontology and Kyoto Encyclopedia of Genes and Genomes enrichment analyses were used to analyze the differentially expressed genes.

### qRT-PCR

Total RNA from cells or tissues was extracted with 1 mL of RNAiso plus (Takara). Then, cDNAs were synthesized with SuperScript II (Takara). A mixture containing 10 μL of All-in-One qPCR reagent (GeneCopoeia), 5 μL of cDNA and 5 μL of primers (forward and reverse, Table 2) was used to perform fluorescence qRT-PCR. The PCR cycling conditions were 50°C for 2 min, 95°C for 10 min, and 40 cycles of 95°C for 15 s and 60°C for 1 min. *GAPDH* was used as an internal reference gene, and relative mRNA expression was analyzed using the 2^-ΔΔCT^ method.

**Table 2:** RT-PCR primer sequence.

**Primers**	**Forward(5'-3’)**	**Reverse(5'-3’)**
Loc490	TTTATGCTTGAGCCTTGA	CTTGCCTGAAATACTTGC
QKI	TAGCAGAGTACGGAAAGACAT	GGGTATTCTTTTACAGGCACAT
E-cadherin	GGGTCTTGCTATGTTGCC	GTTCCGCTCTGTCTTTGG
Vimentin	TCCAAGTTTGCTGACCTCTC	TCAACGGCAAAGTTCTCTTC
N-cadherin	AACTCCAGGGGACCTTTTC	CAAATGAAACCGGGCTATC
MMP-9	TCGAACTTTGACAGCGACAAG	TCAGGGCGAGGACCATAGAGG
GADPH	GTCAACGGATTTGGTCTGTATT	AGTCTTCTGGGTGGCAGTGAT

### Western blot analysis

Cells were lysed in 1 mL of cell lysis buffer (Beyotime Biotechnology). The cell lysate was mixed with 5x loading buffer (Beyotime Biotechnology) and the mixture was heated to 100°C. Then, 50 μg of total protein was separated on a 10% sodium dodecyl sulfate polyacrylamide gel. The protein levels of QKI, E-cadherin, Vimentin, N-cadherin, MMP-9 and α-Tubulin (the internal control) were analyzed using standard techniques with primary antibodies diluted 1:1000. The relative protein levels were calculated based on the strip grey values using ImageJ software.

### Cell proliferation assay and plate colony formation assay

Cells were seeded into 96-well plates at a density of 1×10^3^ cells/well in RPMI-1640 medium (100 μL). After a 24-hour incubation, the cells were transfected with plasmids or short-hairpin RNAs and incubated for another 24 to 72 hours. Cell viability was determined on a VERSAmax microplate reader (Molecular Devices) using the MTS method in accordance with the manual of the CellTiter 96 Aqueous One Solution Cell Proliferation assay (Promega). The cell survival rate was expressed as A/B × 100, where A is the absorbance value from the experimental cells and B is that from the control (untreated) cells.

For the plate colony formation assay, cells (2×10^3^/mL/well) were seeded into six-well plates and cultured in RPMI-1640 medium supplemented with 10% FBS. Colonies were fixed with methanol, stained with crystal violet and scored using ImageJ software.

### Transwell migration and invasion assays

For the Transwell invasion assays, 50 mg/L Matrigel (BD Biosciences) was used to cover the Transwell invasion chamber (Corning). Cells (5×10^5^) were mixed with 100 μL of RPMI-1640 medium containing 2% FBS and seeded into the upper chamber of the Transwell invasion system, while 600 μL of RPMI-1640 medium containing 10% FBS was added to the lower chamber. The chamber was then placed in an incubator for 24 hours. Subsequently, the upper chamber was removed and the medium was discarded. A 10% methanol solution was used for cell fixation and a 0.1% crystal violet solution was used for staining.

The Transwell migration assays were carried out in a Transwell invasion chamber without the covering of 50 mg/L Matrigel. Cells (2×10^5^) were mixed with 100 μL of RPMI-1640 medium containing 2% FBS and seeded into the upper chamber of the Transwell migration system. The other steps were the same as above.

### RNA immunoprecipitation

Cells were resuspended in phosphate-buffered saline and treated with 1 mL of freshly prepared ice-cold lysis buffer on ice for 20 min. Pellets were extracted via centrifugation at 14,000 rpm for 10 min. Then, 2 μg of an anti-QKI antibody and 40 μL of protein A/G beads were added to create a protein suspension, which was incubated for 12 hours at 4°C with gentle rotation. The beads were pelleted through centrifugation at 2,500 rpm for 30 seconds; then, the supernatant was removed and the beads were resuspended in 500 mL of RNA immunoprecipitation buffer. This was repeated for a total of three RNA immunoprecipitation buffer washes, followed by a wash in phosphate-buffered saline. Then, 1 mL of RNAiso was added to extract the total RNA. The remaining processes were the same as those for qRT-PCR.

### *In vivo* xenograft mouse model

Nude mice were inoculated subcutaneously with SGC-7901 cells in three groups: Vector + siControl, Loc490 + siControl, and Loc490 + siQKI (5x10^6^ cells and n = 5/group). Seven days after the inoculation, the tumor length (L) and width (W) were measured with a caliper. Tumor volumes were calculated with the formula L × W^2^ × π/6. All measurements were compared using an unpaired Student’s t-test.

### Statistical analysis

Data were processed with the Statistical Package for the Social Sciences 19.0 (SPSS 19.0, Inc., Chicago, IL, USA). Results are expressed as the mean ± standard deviation (SD). Two-tailed Student’s t-tests were used for comparisons of two groups, while one-way analysis of variance was used for comparisons of three or more groups. A p value < 0.05 was considered statistically significant. *, ** and *** indicate p < 0.05, p < 0.01 and p < 0.001, respectively.

## Supplementary Material

Supplementary Figure 1
