# Supplementary material for: Long non-coding RNA Loc490 inhibits gastric cancer cell proliferation and metastasis by upregulating RNA-binding protein Quaking
Source: Aging (Albany NY). 2020 Sep 15;12(17):17681–93. doi: 10.18632/aging.103876 (PMC7521539; doi:10.18632/aging.103876)
Supplement: Supplementary Figure 1 [file aging-12-103876-s001..pdf]

SUPPLEMENTARY FIGURE

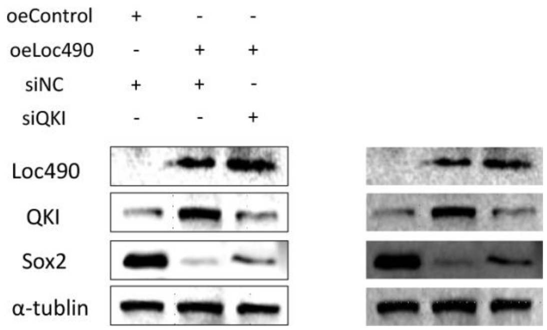

Supplementary Figure 1. The expression of SOX2 were regulated by Loc490/QKI axis.
